# Modeling the public attitude towards organic foods: a big data and text mining approach

**DOI:** 10.1186/s40537-021-00551-6

**Published:** 2022-01-06

**Authors:** Anupam Singh, Aldona Glińska-Neweś

**Affiliations:** Nicolaus Copernicus University, Torun, Poland

**Keywords:** Organic foods, Text mining, Big data, Sentiment analysis, Latent Dirichlet Allocation (LDA), Topic modeling, Machine learning

## Abstract

This study aims to identify the topics that users post on Twitter about organic foods and to analyze the emotion-based sentiment of those tweets. The study addresses a call for an application of big data and text mining in different fields of research, as well as proposes more objective research methods in studies on food consumption. There is a growing interest in understanding consumer choices for foods which are caused by the predominant contribution of the food industry to climate change. So far, customer attitudes towards organic food have been studied mostly with self-reported methods, such as questionnaires and interviews, which have many limitations. Therefore, in the present study, we used big data and text mining techniques as more objective methods to analyze the public attitude about organic foods. A total of 43,724 Twitter posts were extracted with streaming Application Programming Interface (API). Latent Dirichlet Allocation (LDA) algorithm was applied for topic modeling. A test of topic significance was performed to evaluate the quality of the topics. Public sentiment was analyzed based on the NRC emotion lexicon by utilizing *Syuzhet* package. Topic modeling results showed that people discuss on variety of themes related to organic foods such as plant-based diet, saving the planet, organic farming and standardization, authenticity, and food delivery, etc. Sentiment analysis results suggest that people view organic foods positively, though there are also people who are skeptical about the claims that organic foods are natural and free from chemicals and pesticides. The study contributes to the field of consumer behavior by implementing research methods grounded in text mining and big data. The study contributes also to the advancement of research in the field of sustainable food consumption by providing a fresh perspective on public attitude toward organic foods, filling the gaps in existing literature and research.

## Introduction

The most common approach to study customer behavior, including food-related beliefs and emotions, uses the self-reported method, e.g., participants declare themselves their feelings and emotions in interviews or verbal questionnaires [1, 2]. It stands for significant limitation of such studies. While self-report methods have many advantages, they might be affected by biases such as social desirability bias, response bias, and common method bias [3, 4]. Moreover, some problems are very hard to capture; for example, emotions are, by nature, subjectively experienced in a specific context, so their objective assessment is very difficult [5, 6]. Wang et al. [7] and Feizollah et al. [8] argue that state-of-art computational techniques based on machine learning methods can potentially eliminate such methodological issues.

There is growing interest in understanding consumer choices for foods [9, 10]. It is motivated, first of all, by climate change a serious global issue that poses an urgent and perhaps one of the greatest challenges facing humankind [11, 12]; the food industry contributes predominantly to these effects. Agriculture accounts for over a quarter of global greenhouse gas emissions; half of the total habitable land is used for agriculture; it uses 70% of global freshwater withdrawals and causes 78% of waterways’ pollution; 94% of mammal biomass (excluding humans) is livestock [13, 14]. Unsustainable food consumption and its tangible consequences on human health and the environment, direct the public attention to organic foods which are viewed as ecological and healthy [9, 10]. European Union (EU) describes organic production as “an overall system of farm management and food production that combines best environmental practices, a high level of biodiversity, the preservation of natural resources and the application of high animal welfare standards” [15].

The objective of this study is to identify the topics that users post on Twitter about organic foods and to analyze the emotion-based sentiment of those tweets. People find social media platforms convenient to share their experiences, attitude, and opinion. The rise in the number of active users and users’ contents on Twitter delivers the scope for scientific exploration of public attitudes. In the present study, we extract the latent topics on organic foods from Twitter data and analyze the public sentiments about such foods. The study is the first of its kind, in this line of research, to use text mining based on unsupervised machine learning technique, which eliminates the limitations of sample size and subjectivity of conventional methods.

So far, the studies on food consumption have utilized rather subjective traditional research methods, such as questionnaire surveys and interviews [16–19]. Although the body of research on organic food consumption is quite large, the question of why people buy organic food is still not fully understood [16, 20, 21]. Moreover, research shows that while consumers report favorable attitudes toward organic food, they often do not display subsequent buying actions; in other words, there is the intention-behavior gap in the consumer decision-making process regarding organic food purchases [22–24]. Adoption of more objective research methods in the studies on food consumption can help to explain this gap.

To the best of our knowledge, there has been no study analysing organic food consumption behaviour using big data and machine learning techniques. Danner and Menapace [10] explored beliefs regarding organic foods but based on content analysis of online comments posted in news websites and forums. However, our study uses machine learning-based text mining techniques to identify the most discussed themes on organic foods, and analyses also the underlying emotions and sentiments about the same. The study contributes to the field of consumer behavior by implementing research methods grounded in text mining and big data. The study contributes also to the advancement of research in the field of sustainable food consumption by providing a fresh perspective on public attitude toward organic foods using the aforementioned objective methods of data collection and analysis, filling the gaps in existing literature and research.

In the following, the relevant literature on sustainable food consumption, and consumer beliefs and emotions about organic foods are reviewed. Subsequently, the methodology used in the study is explained, followed by results and discussion. Finally, the conclusion of the study is drawn with the limitations and the suggestions for future research.

## Literature review

Organic food production is based on environmentally sustainable techniques excluding chemical or synthetic additives; its ecological nature strongly supports ecosystems and bio-diversity [25–27]. Organic food production and consumption are also considered as animal-friendly and related to fairness and human health [28, 29]. Recent years have brought increased consumers’ interest in organic food. Its global market is valued at 96.7 billion USD and a growing number of countries adopts distinct regulations for its cultivation [30]. Organic food production and trading are among the fastest-growing industries in the food market in Europe, South America, Oceania, and Japan [31].

Customers buy organic foods due to rising food safety concerns [17] and health consciousness [18], but also because of its taste [32–34]. Consumer motivations for choosing organic foods are categorized as related to health, environment, and social consciousness [16]. A rising level of concern for environmental and ecological welfare supports organic food consumption as animal friendly [20, 35] and as a counter-option to conventional food production using chemical, synthetic and genetically modified means [24, 30, 36]. Other non-egotistical factors of organic food consumption include supporting local producers [37] and reflect concern for society and consumer social surroundings [19, 38].

Recent studies suggest that perceived values (utilitarian and hedonic) influence consumer willingness to buy organic foods [39, 40]. It is the perceived value of a product from the multisensory and emotive aspects. The utilitarian value is the assessment of functional benefits and drawbacks of a product or service [41, 42]. In the context of organic foods, the utilitarian value refers to the nutritional quality, purity, safety, and healthy attributes [43]. The hedonic value of organic food refers to the pleasure felt from the taste and the freshness and purity [44].

Concerning emotions influencing sustainable customer behavior, the picture is also very complex. Literature delivers evidence for the consequences of both negative and positive emotions in this regard [45]. Among negative emotions, e.g., guilt is argued to influence sustainable behaviors because of consumer feelings of moral responsibility for the environment [45, 46]. Also, sadness was shown to affect more pro-environmental behaviors, such as giving higher monetary donations to sustainable causes [47]. On the other hand, consumers are likely to engage in sustainable behaviors when they derive positive emotions from the behavior [45, 48]. For example, joy and pride influence intentions to decrease plastic water bottle usage, while optimism can motivate to maintain such behaviors over time [45, 49]. However, positive emotions can also encourage unsustainable consumer behaviors, such as driving gas-powered automobiles [50], so probably also purchasing unhealthy food. Associations between food and emotions influencing food acceptance and preference gained attention in consumer and sensory research e.g. [51, 52].

The review of the literature suggests that the big data and text mining approaches have been implemented by the researchers in different contexts. For example, Wang et al. [53] studied the attitude of the Chinese public toward off-site construction using Sina Weibo data, while Wu et al. [7] explored the attitude of the Chinese public toward municipal solid waste sorting policy; Feizollah et al. [8] studied halal tourism using Twitter data; Philander and Zhong [54] utilized text mining techniques to explore hospitality customers’ attitudes and perceptions using Twitter data; Prameswari et al. [55] applied text mining techniques on online hotel reviews to identify priority tourist destinations in Indonesia; Oliveira et al. [56] used Twitter data to predict stock market behavior. In these studies, it has been argued that text mining techniques used to explore the comments posted, for example, on Twitter, give an insight into public attitudes in the more objective way than traditional research methods. Therefore, addressing the calls for the application of big data and text mining in different fields of research, the present study uses big data and text mining approach to investigate public attitudes about organic foods.

## Research method

**Fig. 1:**
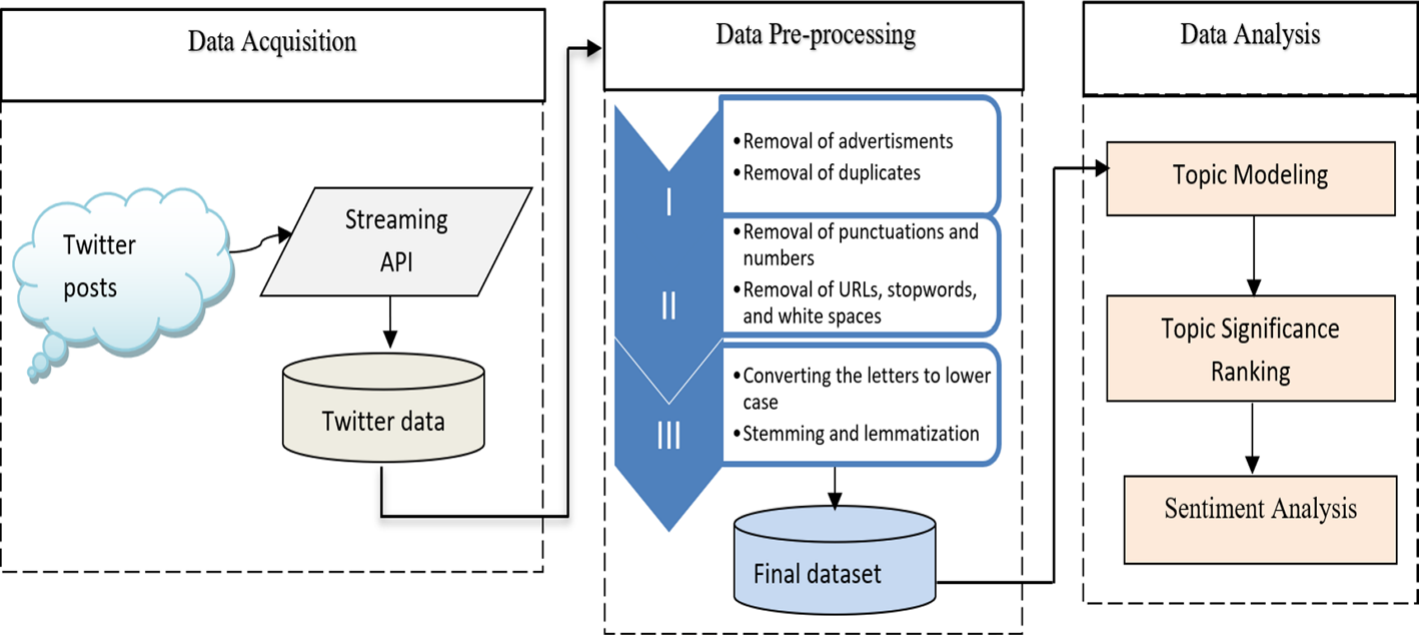
The methodological architecture of the study

This section introduces the methods used in the study, followed by the subsections outlining each stage in detail. Figure 1 depicts the methodological architecture of the study.As shown in Fig. 1, the first step involved data acquisition. Tweets are collected via Twitter APIs using the keyword. In the second step, the tasks of data preprocessing were performed (please refer to the “Data collection” and “Pre-processing” sections for more details). In the third step, the final data obtained from the second step was analyzed. Topic modeling was applied for topic extraction followed by topic significance ranking. In the last step, emotion-based sentiment analysis was performed, wherein the underlying emotions in the tweets were calculated and visualized the overall results.

### Data collection

Twitter posts were extracted for this study. Twitter is a very popular online social network for mass communication and information dissemination [57] and referred to as the gold mine of data [58, 59]. People find Social Networking Sites (SNS) a platform to share their views, opinions, information, and ideas at any time, thereby leaving the scopes for scientific research. The data is easily accessible and is useful for those studying public attitudes, opinions, beliefs, and behaviors [60]. In this study, tweets were obtained by using Twitter Streaming Application Programming Interface (API). R 4.0.4 and Python 3.9 were used for the data extraction, storage, pre-processing, data mining, and result visualization. *Tweepy*, a popular Python library, was used for accessing the Twitter API. The tweets were streamed for the keyword ‘’Organic Food’’. Only tweets written in English were considered, with no geographical restriction. The extracted dataset has the Tweet object which includes fundamental attributes such as ‘created_at’ (the time when this tweet was created), and ‘text’ (tweets), screen_name, and geo-tagged location. However, we have taken only tweets in our analysis.

### Data pre-processing

Social media data is interesting but is unstructured. A piece of meaningful information can be drawn if the quality data is ensured. The unstructured and semi-structured data need pruning to get an insight from it. The quality of Twitter data in this study was ascertained through the framework of accuracy proposed by [61], followed by extensive data pre-processing and cleaning. The accuracy was ascertained keeping in mind the query error, interpretation error, and coverage error during the data collection phase as suggest in the study of [61].

We used Microsoft Excel and R Programming Language for data pre-processing. We adopted the following three steps for data pre-processing. Denoising of data was done in the first step. In here, advertisements, duplicate data, and irrelevant posts were deleted. In the second step, stop words, URLs, symbols, and the words that appear frequently and do not contribute to sentiment analysis were also removed. We employed the ‘tm’ package to remove such contents from the text data. Stop words, such as “is”, “the”, “an”, “how”, and “what”, were eliminated as these words do not contain relevant information. Similarly, URLs and numbers also do not contain any information, thus were eliminated. Finally, in step 3, words were converted into the lower case before stemming and lemmatization was done. Thus, the cleaned data obtained was considered in the final analyses of the study.

### Topic modeling

Latent semantic analysis (LSA), probabilistic latent semantic analysis (pLSA), and LDA are the three most commonly used topic modeling techniques [62]. We adopted LDA in this study for the following reasons. LSA is an effective dimensionality reduction technique, which can achieve significant compression in large collections while capturing some aspects of basic linguistic notion. However, the drawback of LSA is its inability of fitting a model to data to represent the documents into multiple topics. Although the pLSA approach solves the representation challenge in LSA, it cannot generalize to unseen documents due to the lack of a well-defined generative model. The method is also prone to overfitting [63], which is a serious issue, whereas LDA is capable of performing dimensionality reduction, semantic annotation, mixture modeling, and generalization ability without such major issues [62]. Since this study is based on the cross-sectional research design, analysis of the temporal pattern of associated topics and topic correlation is out of the scope of this paper. Therefore, the topic modeling techniques like ‘Topics over Time’, ‘Dynamic Topic Model’ and ‘Pachinko Allocation Model’ are ruled out. Thus, LDA-based topic modeling suits well for achieving the objectives of this study.

**Fig. 2:**
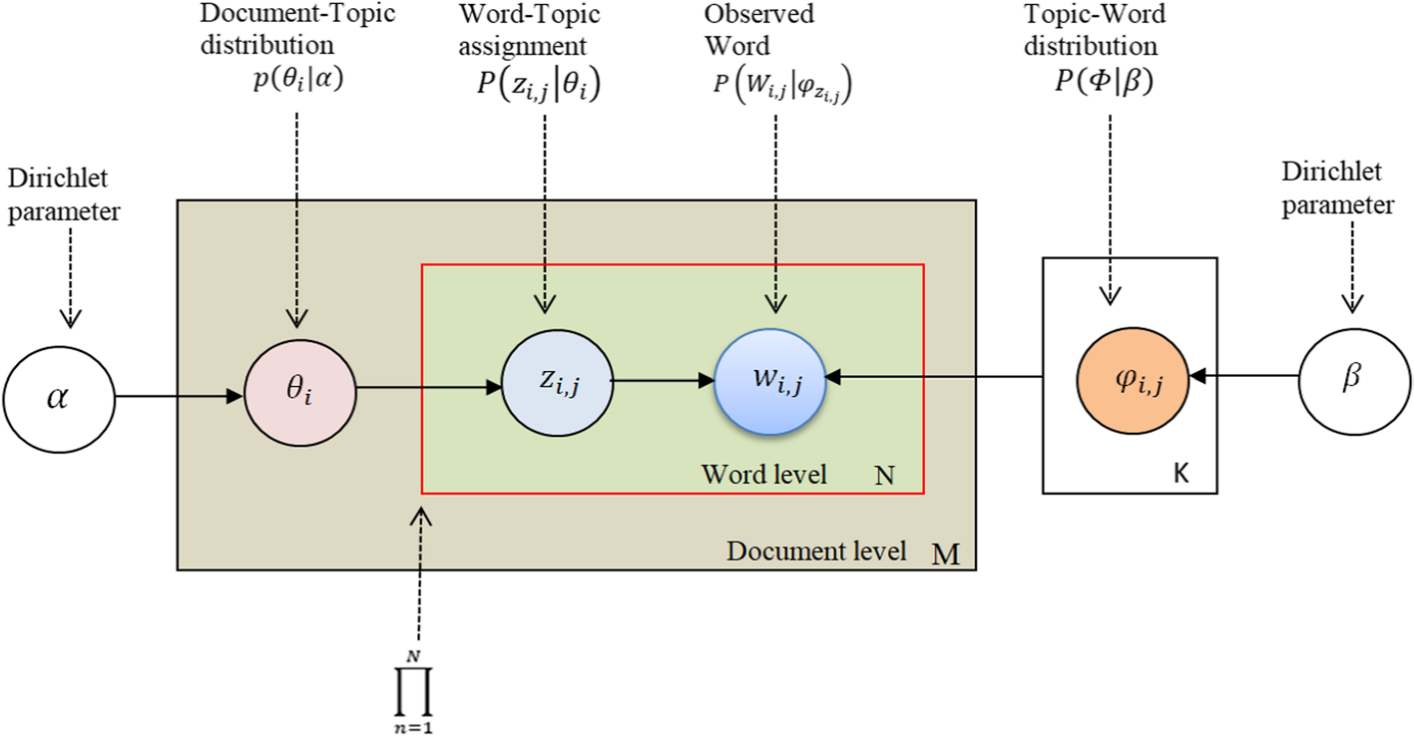
Graphical model representation of LDA

LDA is a sophisticated unsupervised machine learning technique for identifying the key topics from large textual data [63]. It has been widely used in the field of natural language processing (NLP) among several topic modeling algorithms [7, 64]. It is a three-stage Bayesian probabilistic model. The model gives the topic of each document in the documents (D) with a distribution over the topics (K), and each topic is a multinomial distribution over words (W) in the corpus. The statistical model adopts the following three stages generative process (Fig. 2) for a corpus D of M documents with length N_i_ of each; α is the Dirichlet parameter of per-document topic distributions, and β is the Dirichlet parameter of per-topic word distribution. And, θ_i_ and φ_*k*_ denote the topic distribution for the document *i* and the word distribution for topic *k*, respectively.

Based on the definition, LDA assumes the following generative processes for each tweet (w) in a corpus (D):

- Choose N ~ Poisson (ξ), where N denotes the length of documents;
- Draw a topic distribution, choose ${\theta }_{d}$ ~ Dir(α), where α is the parameter of the Dirichlet prior on the per-tweet topic distributions; and
- For each of N words ${w}_{n}$:
- Choose a topic ${z}_{n}$ ~ Multinomial (θ); and
- Choose a word ${w}_{n}$ from $p\left(\left.{w}_{n}\right|{z}_{n},\beta \right)$, a multinomial probability conditioned on the topic ${z}_{n}$.

Step 1 indicates the selection of number of words N for a document, based on a Poisson distribution. The dimensionality k of the Dirichlet distribution is assumed known and fixed. Step 2 refers to the probability that the dimension (topic) occurs and is represented as a k dimensional random variable θ in a given document (tweet). According to probability density function, it can be expressed as follows:

1 $$p\left(\left.\theta \right|\alpha \right)=\frac{\Gamma \left({\Sigma }_{i=1}^{k}{\alpha }_{i}\right)}{{\Pi }_{i=1}^{k}\Gamma \left({\alpha }_{i}\right)}\prod_{i=1}^{k}{\theta }_{i=1}^{{\alpha }_{i}-1}$$

The variable θ can take values in the (k − 1)-simplex ${\theta }_{i},\cdots ,{\theta }_{i-1}^{k}>0, \sum_{i=1}^{k-1}{\theta }_{i}<1, \sum_{i=1}^{k}{\theta }_{i}=1,$ where the parameter α is a k-vector with components ${\alpha }_{i}>1$, and $\Gamma \left(x\right)$ is the gamma function. Equation 1, a probability density function of Dirichlet distribution, is a conjugate prior for the multinomial distribution, which facilitates the development of inference for Eq. (2).

In step 3, the given parameters α and β, the joint distribution of a topic mixture θ, a set of N topics z, and a set of N words w is expressed as:

2 $$p\left(\theta ,z,\left.w\right|\alpha ,\beta \right)=p\left(\left.\theta \right|\alpha \right)\prod_{n=1}^{N}p\left(\left.{z}_{n}\right|\theta \right)p\left(\left.{w}_{n}\right|{z}_{n},\beta \right)$$

where $p\left(\left.\theta \right|\alpha \right)$ refers to tweet-level (i.e., document-level) parameters, while the other terms refer to word-level parameters. And, $p\left(\left.{z}_{n}\right|\theta \right)$ is ${\theta }_{i}$ for the unique i where ${z}_{n}^{i}=1$.

We can obtain the marginal distribution of a tweet in our corpus by using Eq. (3):

3 $${p}_{\left(\left.w\right|\alpha ,\beta \right)=\int p\left(\left.\theta \right|\alpha \right)}\left(\prod_{n=1}^{N}\sum_{{z}_{n}}p\left(\left.{z}_{n}\right|\theta \right)p\left(\left.{w}_{n}\right|{z}_{n},\beta \right)\right)d\theta$$

Finally, the probability of a corpus can be obtained by taking the product of the marginal probabilities of single documents (i.e., tweets, in our case):

4 $$P\left(\left.D\right|\alpha ,\beta \right)=\prod_{d=1}^{M}\int P\left(\left.{\theta }_{d}\right|\alpha \right)\left(\prod_{n=1}^{{N}_{d}}\sum_{{z}_{{d}_{n}}}P\left(\left.{z}_{{d}_{n}}\right|{\theta }_{d}\right)P\left(\left.{w}_{{d}_{n}}\right|{z}_{{d}_{n}},\beta \right)\right)d{\theta }_{d}$$

where the term ${\theta }_{d}$ is document-level variable assumed to be sampled per tweet. The variables ${z}_{{d}_{n}}$ and ${w}_{{d}_{n}}$ are word-level variables and are sampled once for each word in each tweet. The graphical representation is illustrated by Fig. 2 [63].

The model statistically discovers the group of topics that best explains the observed words in the entire documents. We utilized ‘*topicmodels’* library of the R package for this task. *LDAvis*, a web-based interactive visualization tool, was also utilized for the visualization of topic models.

### Sentiment analysis

Sentiment analysis (also known as opinion mining or emotion AI) is located at the heart of NLP, text mining, and computational linguistics. It measures the subjective information extracted from textual data. In this era of the internet and social media, sentiment analysis has gained much popularity among researchers from different domains for its applicability in a wide range of fields such as psychology, linguistics, public health, and business management [65]. In the current study, the word-level sentiment analysis was performed. NRC Emotion Lexicon was used to list the words and their association with eight basic emotions (trust, fear, surprise, sadness, disgust, anger, anticipation, and joy) and two sentiments (negative and positive). *Syuzhet* package was utilized to analyze the underlying sentiments. The algorithm calculates the presence of eight different emotions and sentiments in the text dataset. It assigns each token (i.e., word) in the corpus a score corresponding to each emotion and sentiments; ‘1’ if it is associated, otherwise ‘0’. Thus, a data frame containing sentiment scores is obtained, where each row represents a word and columns represent eight different emotions and two sentiments.

## Results

The data for this study was collected from Twitter. The tweets posted between January 10, 2021 and March 7, 2021 was extracted using Python. A total of 43,724 tweets were collected. However, after removing the duplicates, 41,009 tweets are processed for further analysis. Before the topic analysis, it is necessary to evaluate the models.

### Topic models’ evaluations

LDA topic modeling is based on probabilistic inference; hence, requires a huge amount of data and tuning to get reasonable results [66]. We used two measures- perplexity and topic coherence- to evaluate the best underlying topics in the corpus. Perplexity is defined as the inverse probability of the test set, normalized by the number of words. It is an intrinsic evaluation metric and is measured as the normalized log-likelihood of a test set data. It is a general quality measure for the entire model [63], and captures how well a probability model predicts the sample for new data it has not seen before.

For training and validation of the model, the data was divided into two sets. 80% of data (in-sample set) was employed to train the LDA model and the remaining held-out set (validation set) of 20% data was employed for model validation. Table 1 shows the results of perplexity scores of 15 topics. The model with nine topics scores the minimal validation perplexity of 237.5, indicating that the model with eight topics is more reliable than the other models. The perplexity validation does not test the semantic similarity between words in a topic. Therefore, perplexity optimization cannot yield human interpretable topics [67]. Thus, this test alone may not be an adequate measure for predicting the best model. This limitation of perplexity warrants a test for topic coherence.

**Table 1:** Validation perplexities under the different numbers of topics

Number of topics	Validation perplexity	Number of topics	Validation perplexity	Number of topics	Validation perplexity
1	290.5	6	247.9	11	244.2
2	269.1	7	241.1	12	256.9
3	276.4	8	237.5	13	251.9
4	253.7	9	239.4	14	270.7
5	245.6	10	243.3	15	267.2

**Fig. 3:**
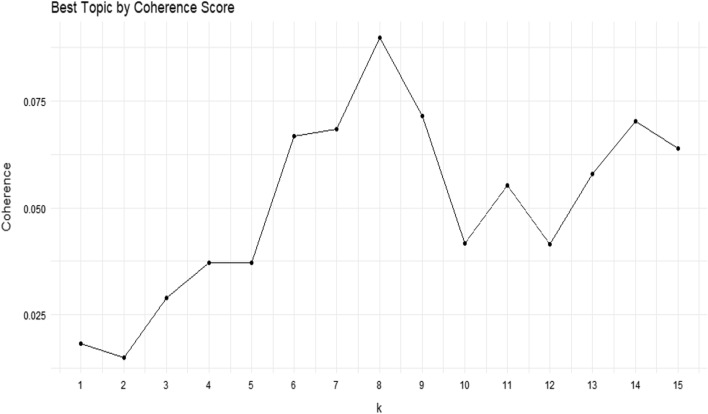
Best topic by coherence score

Coherence is another method of evaluation of a topic model. It measures the degree of semantic similarity between high-scoring words in the topic. The coherence score is calculated based on pairwise scores of the top n-frequently occurring words in a topic. There are different coherence measures discussed in the literature. However, we used the CV measure, the best measure to compare different topic models based on their human interpretability [68]. The measurement helps to distinguish between topics that are an artifact of statistical inferences, and semantically interpretable. Figure 3 shows the coherence score for the different number of topics. A model with the highest coherence score before flattening out or a major drop is considered as the best model. To our surprise, both the tests confirm the presence of eight topics in our data. Therefore, we picked a model with eight topics (k = 8).

### LDA model

Based on the results of perplexity and coherence scores, we ran an LDA model of 8 topics with 12 terms. Topics are labeled based on the identification of a logical connection between most the frequent terms of the topic. Table 2 presents the topic labels with their key terms. Topic 1 and Topic 2 are about the United States (US) politics as tweets were collected between January 10 and March 7, 2021, which is very close to an incidence in the US. Jacob Chansley, a Capitol Hill attacker, demands all-organic foods inside the prison [69]. This incident has led to a huge inflow of Twitter posts related to US politics and organic foods. Topic 3 discusses organic food in connection with food additives such as pesticides and chemicals. The topic is composed of both positive perceptions (i.e., organic food has no additives) and negative or skeptical views (i.e., organic food is not free from additives). It can be understood with the tweets: “*Deceptive Labels on Food. Making sure you understand the terms that are used*”, *“what if they are not using organic eggs feeding the chickens GMO FOOD”.* The key terms are “people”, “health”, “grown”, “eating”, “chemicals”, etc. Topic 4 is about the seasonality of organic foods. It emphasizes on benefits of choosing seasonal organic foods. The key terms are “seasonal”, “package”, “vitality”, and “better”.

**Table 2:** Topics listing top 12 key terms extracted from LDA model

Topic id	Terms	Label
Topic 1	food, organic, jail, judge, Capitol, horns, get, hunger, riot, ordered, fur, strike	US politics (Attack on Capitol Hill)
Topic 2	organic, food, get, jail, man, white, prison, guy, judge, horns, privilege, vacation	US politics (Capitol attacker’s demand for organic food)
Topic 3	organic, food, people, consumption, health, vegan, grown, eating, fed, pesticides, chemicals	Authenticity
Topic 4	food, organic, month, seasonal, three, spring, package, survival, vitality, bundle, chakra, better	Seasonality
Topic 5	organic, food, plant-based, vegan, diet, ecological, refreshing, vegetables, health, future, good	Plant-based diet
Topic 6	organic, food, plant, local, fresh, natural, farming, agriculture, save, planet, impact, world	Saving the planet
Topic 7	farming, choose, harvest, latest, technology, crops, labels, standards, agriculture, supply-chain, organic, focus	Organic farming and standardization
Topic 8	online, grocery, demand, local, store, orders, delivery, affordable, save, COVID19, planet, markets	Food delivery

Topic 5 discusses the importance of shifting toward plant-based foods. Some of the key terms are “plant-based”, “vegan”, “diet”, and “vegetables”. Topic 6 is about saving the planet by natural and local farming which is suggested by terms such as “natural”, “planet”, “save”, “impact” and “world”. Topic 7 highlights the need for technology and standardization in organic farming and supply-chain. The key terms are “farming”, “organic”, “technology”, and “standards”, etc. The last topic discusses the surge in online orders of organic foods and delivery systems, presumably caused by the COVID-19 pandemic.

**Fig. 4:**
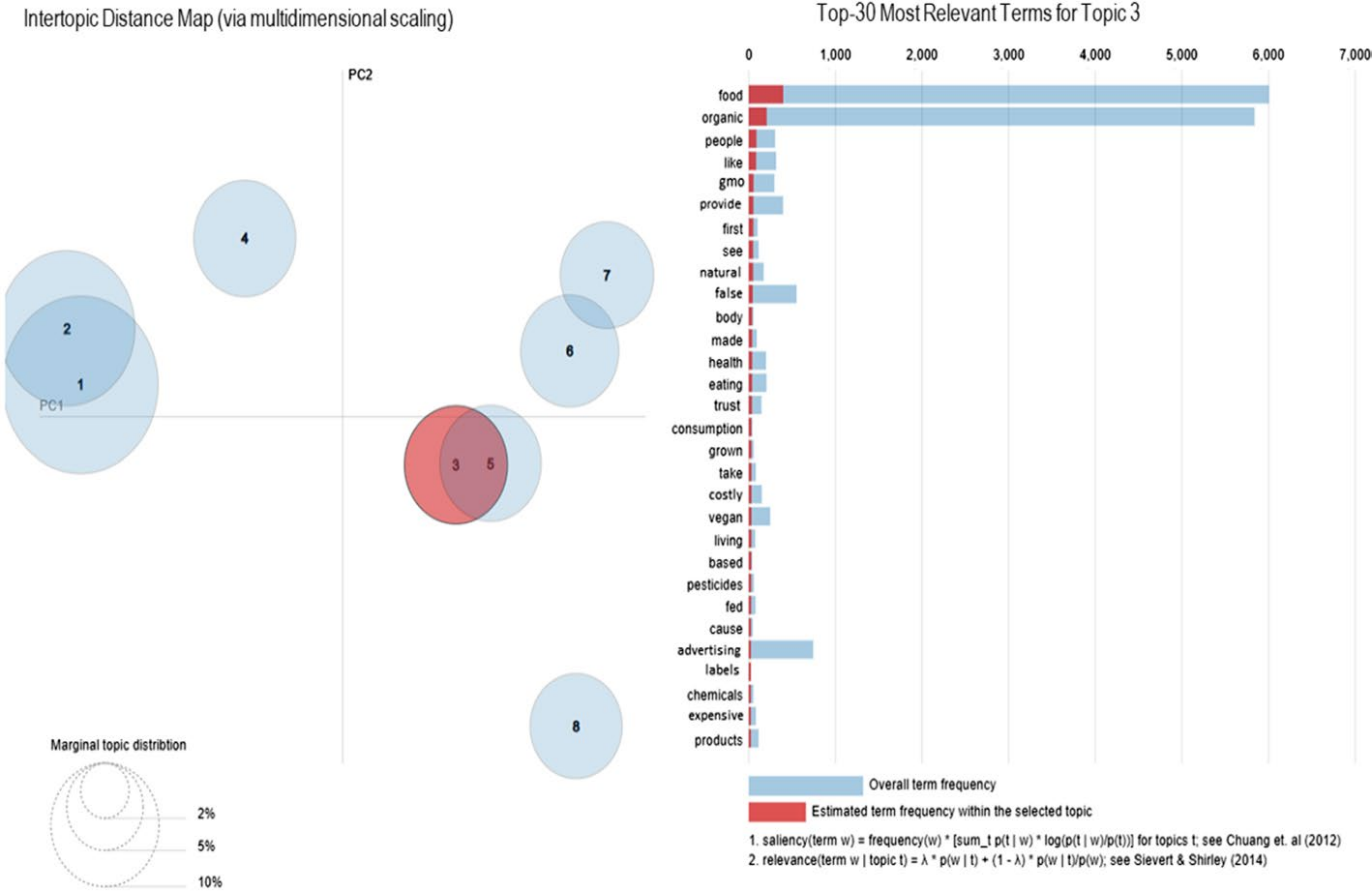
LDAvis visualization for Topic 3

The visual display map of topic models produced using LDAvis highlights the topic with its top 30 most relevant terms. The display map of Topic 3 is shown below as Fig. 4, and the visual display map of the remaining five topics on organic foods is appended at the end of the paper. The visualization provides the global view of topics with the terms highly associated with the individual topic.

In the visual display map on the left side, the distance between the circles indicates the topic similarity (or dissimilarity), i.e., the closer the circles, the more similar the themes. And, the size of the circle depicts its prevalence. The horizontal bar chart on the right side of the visualization depicts the terms most relevant to the selected topic.

As per the display map (Fig. 4), Topic 1 and Topic 2 are similar to each other but differs much from other topics. Similarly, Topic 3 and Topic 5 are close to each other. Topic 8 is away from other topics and refers the unrelatedness as it discusses organic food demands and online deliveries during the COVID-19 pandemic.

### Topic significance ranking

We assessed topic quality through topic significance ranking (TSR) scores based on the criteria proposed by [70]. TSR is a novel unsupervised analysis of Probabilistic Topic Models (PTM). We applied a 4-phase Weighted Combination decision strategy to evaluate the inferred topics of PTM to evaluate their semantic significance. Topic significance is obtained by combining the information from the “multi-criteria measures” to form a single index of evaluation based on a “Weighted Linear Combination” (WLC) decision strategy. WLC is a widely used technique in the area of multi-criteria decision analysis. WLC assesses each topic by the following formula:

5 $${A}_{k}=\sum_{m=1}^{{N}_{m}}{\Psi }_{m}{S}_{m,k}$$

where ${N}_{m}$ is number of different measures, ${\Psi }_{m}$ refers the weight of the measure in the total score, and ${S}_{m,k}$ is the score of ${k}$ th topic for the ${m}$ th measure.

A 4-phase weighted combination approach was implemented. ${S}_{m,k}$ and ${\Psi }_{m}$ scores were to be computed on different scales and criteria; therefore, measures are standardized before combination.

#### Phase 1: Standardization procedure

In the first phase, “standardization procedures” are performed to transfer each distance measure from its true value into two standardized scores (relative score of the distances and weight value).

To compute both the scores ${S}_{m,k}$ and weights ${\Psi }_{m}$, two standardized procedures are used for each of the criteria. The first, score is re-scaled based on the weight of each score against the total score overall topics and is expressed in Eq. (6). The second, score range procedure is followed based on the minimum and maximum values as scaling points for standardization and is given by Eq. (7).

6 $${C}_{{1}^{k}}^{m}={C}_{k}^{m}\times \frac{{\Sigma }_{J=1,j\ne k}^{k}{C}_{j}^{m}}{{\sum }_{J=1}^{k}{C}_{j}^{m}}$$

7 $${\mathrm{C}}_{{2}^{\mathrm{k}}}^{\mathrm{m}}=\frac{{\mathrm{C}}_{\mathrm{k}}^{\mathrm{m}}-{\mathrm{C}}_{\mathrm{min}}^{\mathrm{m}}}{{\mathrm{C}}_{\mathrm{max}}^{\mathrm{m}}-{\mathrm{C}}_{\mathrm{min}}^{\mathrm{m}}}$$

where ${C}_{min}^{m}$ and ${C}_{max}^{m}$ are the minimum and maximum distance values measured by the distance measure *m* under the criterion *C*, and $m\in \left\{KL,COR, COS\right\}$; KL-Kullback–Leibler (KL) Divergence, COR-Correlation Coefficient, and COS- Cosine Dissimilarity are three distance measures.

#### Phase 2: Intra-criterion weighted linear combination

To compute the rank significance, different measures are combined into a single figure. In this phase, topic ranking performs a WLC of the standardized score of the distance measures. The WLC score of criteria *C* for topic *k* is given by Eq. (8).

8 $${s}_{k}^{C}=\frac{{C}_{k}^{KL}+{C}_{k}^{COR}+{C}_{k}^{COS}}{3}$$

where ${C}_{k}^{KL}$, ${C}_{k}^{COR}$, and ${C}_{k}^{COS}$ are the standardized scores of the three distance measures.

#### Phase 3: Inter-criterion Weighted Combination

In phase 3, a Weighted Combination (WC) is done over the scores computed in phase two. Two different WC approaches are used to combine the scores and weights. The first approach is based on Eq. (6) and applies the standardized score of the background criterion as a weight for the Uniformity (u) and Vacuousness ($\nu$).

9 $${\widehat{S}}_{k}={\widehat{s}}_{{1}^{k}}^{b}\left({\Psi }_{u}{S}_{{1}^{k}}^{u}+{\Psi }_{v}{S}_{{1}^{k}}^{v}\right)$$

where ${\Psi }_{u}$ and ${\Psi }_{v}$ are the weights of the Uniformity and Vacuousness criteria in the score, respectively; ${\widehat{S}}_{{1}^{k}}^{b}$ is the weight of the topic background.

The second approach is performed based on the score range standardization procedure (Eq. 7). This is done by using the WLC in Eq. (5) as below:

10 $${\Psi }_{k}={\Psi }_{u}{S}_{{2}^{k}}^{u}+{\Psi }_{v}{s}_{{2}^{k}}^{v}+{\Psi }_{b}{s}_{2}^{b}k$$

#### Phase 4: Topic Significance Score

The final rank is computed using the total topic score from Eq. (9) and the normalized weight from Eq. (10). Thus, the final rank of the topic is computed by:

11 $$TS{R}_{k}={\Psi }_{k}\times {\widehat{S}}_{k}$$

A significance ranking algorithm was applied on the topics discovered by LDA with K set to 8. This technique has the potential to properly rank the topic based on its true significance. The rank of a topic depends on its distribution of background words. A topic with higher TSR describes the underlining themes better and presents the meaningful class semantics. Table 3 presents the topic significance rank index. The topics are ordered by their rank.

**Table 3:** Topic significance rank

Topic id	Topic label	$\widehat{S}$	$\widehat{\Psi }$	TSR	Rank
Topic 5	Plant-based diet	21.3	0.8	17.0	1
Topic 3	Authenticity	20.7	0.8	16.6	2
Topic 4	Seasonality	19.8	0.8	15.8	3
Topic 7	Organic farming and standardization	18.5	0.8	14.8	4
Topic 6	Saving the planet	16.3	0.8	13.0	5
Topic 2	US politics (Capitol attacker’s demand for organic food)	15.7	0.8	12.6	6
Topic 8	Food delivery	14.8	0.7	10.4	7
Topic 1	US politics (Attack on Capitol Hill)	14.5	0.7	10.2	8

According to our *LDAvis* results, Topic 1 and Topic 2 are the most prevalent topics. However, these two topics receive low significance ranks. Topic 5 (Plant-based diet), Topic 3 (Authenticity), and Topic 4 (Seasonality) are the three most significant topics.

### Sentiment results

**Fig. 5:**
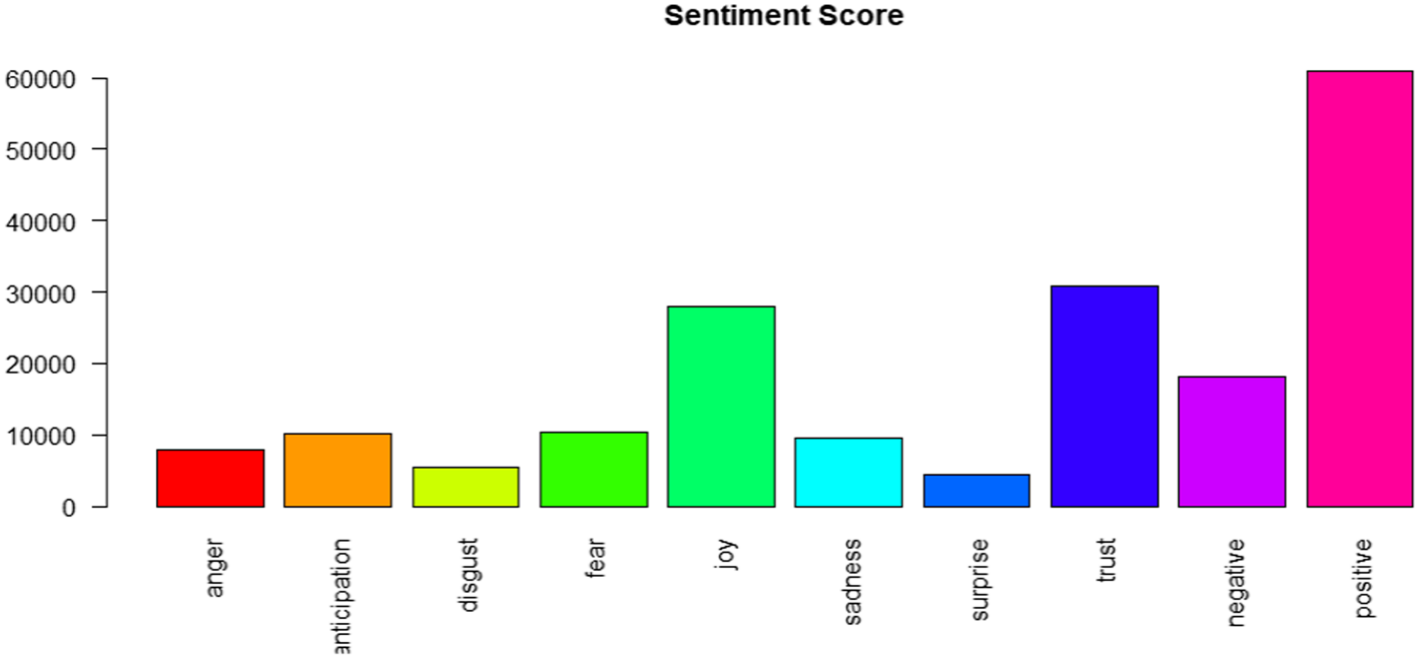
Public sentiments toward organic foods

NRC lexicon enabled us to classify eight different emotions in addition to positive and negative sentiments. The negative emotions include fear, anger, disgust, and sadness, while positive emotions include joy, trust, anticipation, and surprise. The results of sentiment analysis show that public views toward organic foods are mostly positive (see Fig. 5). The results also show the presence of negative emotions but in a lower proportion as compared to positive ones. The frequent positive words for organic food are “healthy,” “safe,” “fascinating’’, “eat,” “perfect,” “vitality,” “aroma,” and “delicious,” etc. Joy and trust are two prominent emotions expressing positive emotions about organic foods with a total sentiment score of more than 30,000 for each.

The frequent negative words are “unnatural’’, “waste,” “doubt,” “junk,” “ridiculous,” “fake,” and “unbelievable,” etc. The keywords suggest that people are suspicious about the authenticity of organic foods, and express negative emotions. Some of the tweets are: *“Only 4–6% of the food is organic. The rest is produced by a few major food processors and full of chemicals*,” *“Can I sue the food place for false advertising?” and “All food is organic*. *Inorganic matter can't be digested*.” Therefore, the fact cannot be ignored that not everyone is convinced about organic claims, i.e., people are still skeptical about organic foods available in the markets being natural, healthy, and chemical or pesticide-free. However, the entire negative emotions in the results are not caused by the public distrust alone. It contains the negative emotions about the US riot and politics over organic foods (“insurrection,” “disgraceful,” “riot,” “attack,” etc.).

## Discussion

The quality of output of a machine learning model depends on the purity of the data and the training of the model. In the present study, the data passes through rigorous data cleaning and pre-processing followed by model tuning and evaluations. Topic evaluation results indicated the presence of eight topics reflecting public attitude on organic foods. Except for Topic 1 and Topic 2, which discuss organic foods linking a specific incidence in the USA, all other topics highlight various aspects related to organic foods.

The majority of previous studies on organic food consumption were based on traditional, self-reported methods. These studies focused, for example, on consumers’ motivations to buy organic foods, such as health, environment, and social consciousness [16]. The only study based on content analysis of online comments about organic foods [10] revealed 65 beliefs that the authors structured into 22 themes of a wide breadth, from ‘price’ to ‘food security’. The authors structured them into four main categories: ‘product’, ‘food system’, ‘authenticity’, and ‘production’. Our study contributes to this line of research by providing further structuring of public attitudes on organic foods based on objective techniques of big data and text mining. Moreover, based on the topic significance rank test, we have identified top the three most significant topics, i.e., plant-based diet, authenticity, and seasonality. The other significant themes related to organic food are organic farming and standardization, saving the planet, and food delivery. Our findings suggest also that people are quite enthusiastic about shifting to plant-based food. They believe that a plant-based diet can contribute to reducing greenhouse gas emissions. Dedicating natural resources such as land and water for growing plant-based foods can save the planet (Topic 6). Authenticity is another interesting theme. Under this theme, tweets indicated that a large group of people advocate organic food for being natural, healthy, non-GMO, and chemical and pesticide-free, while another group is skeptical about these claimed features of organic foods. On the *‘*seasonality,’ people tweeted about the benefits of eating seasonal foods. Organic food eaters find seasonal foods fresh, tasty, and healthy [71].

People in their tweets also highlighted the need for organic farming using the latest technology and standardization of food products and processes (Topic 7). The standards also aim to avoid “false organic claims.” Unfortunately, there are differences in standards across the countries. Further, Topic 8 discusses online delivery services and the availability of organic foods in local stores and markets. Online food retailers have witnessed “unprecedented demand” for organic products during COVID19 lockdowns [72, 73]. Whenever there is a health scare (like SARS/COVID19), people look for ways to disease prevention and boost their health. According to a study by Tastewise, an AI-powered intelligence startup, consumers’ mention of ‘immunity’ in online food searches rose 27% between February 2019 and March 2020 [73]. As people, especially in the outbreaks, search for healthy and safe foods for their at-home families, organic foods are proving to best food choice [74].

Surprisingly, our results do not report any theme on price. It is contrary to the findings of most of the survey research [34, 75–78], which reports organic food is more expensive than conventional food, which is a major consumer concern. However, results of [10], based on content analysis of online comments about organic food posted on news websites and forums, report two positive price beliefs- namely, ‘organic products are worth the price premium’ and ‘organic products are not (much) more expensive than conventional products.’ Thus, when it comes to health and environmental benefits from the products, consumers pay less attention to the price [79]. This might be a likely reason for the absence of a prominent theme on the price in our results. Whereas, in line with our findings, ‘authenticity’ was found to be a most discussed theme in the study of [10].

Our sentiment analysis results show that public sentiment is predominantly positive toward organic foods. However, a small proportion of people has negative sentiments too. This result corroborates the findings of [80]. In their study, [80] also found a high relevance for positive sentiment valence than the negative valence analyzed from comments made on organic food videos on YouTube. Positive sentiments can be attributed to hedonic (taste, freshness, appearance, aroma, etc.) and utilitarian (nutritional contents, healthfulness, ecological welfare, healthfulness, and purity, etc.) values. The research from a consumer cognition perspective also suggests the emotions about organic foods are the outcome of belief held for its healthiness and safety, ecological consequences, as well as a role in supporting local economies. As per our findings, negative emotions about organic foods are mainly due to public skepticism about organic claims.

## Conclusion

Since the consumer behavior towards organic food is changing rapidly due to awareness of environmental degradation, health hazards, and other related issues, this research started with an aim to answer the same existing question- how does the public view organic foods? Numerous studies have been conducted in the past. The majority of them are based on surveys and interviews, which are subject to various limitations such as social desirability bias, a response bias, and common method bias. In this study, we collected public posts from Twitter and utilized text mining techniques to extract the most discussed topics and sentiments towards organic foods. People willingly publish their opinions online on Twitter, which is free of an external bias. The findings obtained by utilizing the big data from social media platforms for text mining, provide relatively objective and verifiable results.

The present study makes academic as well as practical contributions. From an academic perceptive, this is the first of early researches to study the public’s attitude and sentiments towards organic foods using big data and text mining approaches. Also, this study adds to the existing literature by providing a broader and richer understanding of public perspectives on organic food consumption. The study explored public attitude on organic foods and identified underlying different emotions as opposed to just positive or negative sentiments. The results indicated the presence of negativity among the public mainly due to distrust in the authenticity of organic claims. Therefore, eliminating the distrust among the consumers may boost the growth of the organic food market. Thus, from a practical perspective, our findings can help policy-makers and certification bodies enhancing the communications about organic foods, thereby developing trust and positivity.

This research has some limitations. First, the public posts were extracted from Twitter during a specific time period. Second, users of Twitter may not cover all age groups as the elderly people may not be familiar with the internet and social networking sites. Third, only posts in English were analyzed. Therefore, for large and diverse sample data, future research should collect the data from different social networking sites for a larger time span or in different time intervals. Future research, in this direction, is encouraged to perform a topic modeling and sentiment analysis of texts in other languages to validate our findings. Last, a lexicon-based approach was used in the present study, which may fail to identify some human expressions such as sarcasm and irony.

## Data Availability

The dataset used in this study can be obtained from the corresponding author on reasonable request.

## Appendix A. Topic visualizations

A1: LDAvis visualization for Topic 4.

A2: LDAvis visualization for Topic 5.

A3: LDAvis visualization for Topic 6.

A4: LDAvis visualization for Topic 7.

A5: LDAvis visualization for Topic 8.

## Abbreviations

LDA: Latent Dirichlet Allocation
SNS: Social Networking Site
API: Application Programming Interface
NLP: Natural language processing
TSR: Topic significance ranking
